# The Parasitic Worm Product ES-62 Targets Myeloid Differentiation Factor 88–Dependent Effector Mechanisms to Suppress Antinuclear Antibody Production and Proteinuria in MRL/*lpr* Mice

**DOI:** 10.1002/art.39004

**Published:** 2015-03-27

**Authors:** David T Rodgers, Mairi A McGrath, Miguel A Pineda, Lamyaa Al-Riyami, Justyna Rzepecka, Felicity Lumb, William Harnett, Margaret M Harnett

**Affiliations:** 1 University of GlasgowGlasgow, UK; 2 University of StrathclydeGlasgow, UK

## Abstract

**Objective:**

The hygiene hypothesis suggests that parasitic helminths (worms) protect against the development of autoimmune disease via a serendipitous side effect of worm-derived immunomodulators that concomitantly promote parasite survival and limit host pathology. The aim of this study was to investigate whether ES-62, a phosphorylcholine-containing glycoprotein secreted by the filarial nematode *Acanthocheilonema viteae*, protects against kidney damage in an MRL/*lpr* mouse model of systemic lupus erythematosus (SLE).

**Methods:**

MRL/*lpr* mice progressively produce high levels of autoantibodies, and the resultant deposition of immune complexes drives kidney pathology. The effects of ES-62 on disease progression were assessed by measurement of proteinuria, assessment of kidney histology, determination of antinuclear antibody (ANA) production and cytokine levels, and flow cytometric analysis of relevant cellular populations.

**Results:**

ES-62 restored the disrupted balance between effector and regulatory B cells in MRL/*lpr* mice by inhibiting plasmablast differentiation, with a consequent reduction in ANA production and deposition of immune complexes and C3a in the kidneys. Moreover, by reducing interleukin-22 production, ES-62 may desensitize downstream effector mechanisms in the pathogenesis of kidney disease. Highlighting the therapeutic importance of resetting B cell responses, adoptive transfer of purified splenic B cells from ES-62–treated MRL/*lpr* mice mimicked the protection afforded by the helminth product. Mechanistically, this reflects down-regulation of myeloid differentiation factor 88 expression by B cells and also kidney cells, resulting in inhibition of pathogenic cross-talk among Toll-like receptor–, C3a-, and immune complex–mediated effector mechanisms.

**Conclusion:**

This study provides the first demonstration of protection against kidney pathology by a parasitic worm–derived immunomodulator in a model of SLE and suggests therapeutic potential for drugs based on the mechanism of action of ES-62.

Systemic lupus erythematosus (SLE) is characterized by high titers of autoantibodies, typically against nuclear antigens. These autoantibodies generate immune complex–mediated inflammation in the kidneys, skin, joints, and cardiovascular system, with glomerulonephritis being a major contributor to resultant morbidity 1. Inflammation in the kidney is driven by cross-talk among immunoglobulin (Fc receptor [FcR]), complement, and Toll-like receptors (TLRs), resulting in the production of cytokines and infiltration of proinflammatory cells, which perpetuate chronic inflammation and organ damage 1–3. Studies in interleukin-23 (IL-23)–deficient mice suggest that the IL-23/IL-17 axis promotes such kidney inflammation 4, and, perhaps reflecting this, expanded populations of Th17- and IL-17–producing CD3+CD4−CD8− T cells are observed in the kidneys of both lupus-prone mice and patients with SLE 5. Moreover, IL-17 has been reported to act in concert with BAFF to promote B cell survival and (auto)antibody production 4–6. Consistent with the central role of B cells in the pathogenesis of SLE, increased expression of BAFF correlates with disease activity in SLE, and overexpression of BAFF promotes SLE-like pathology in mouse models, even in the absence of T cells. Specific targeting of this cytokine has proved effective in suppressing pathology, in both mouse models and human patients 3, and indeed, belimumab (an anti-BAFF monoclonal antibody) is the first SLE-specific treatment to be granted Food and Drug Administration approval in the past 50 years, although disappointingly, disease activity was reduced only in a limited number of patients during phase III trials 3,7.

Autoimmune inflammatory disorders appear to be increasingly prevalent in the developed world. As suggested by the hygiene hypothesis 8, this may reflect reduced exposure to infection, particularly by parasitic helminths (worms), which would normally shape and balance immune responses to limit pathology and promote tissue repair 9,10. Consistent with this notion, in experimental models of autoimmune disease, infection with helminths was shown to be protective 9,10, and this has generated interest in the potential for exploiting worm-based immunomodulation for the treatment of these inflammatory disorders in humans. Although clinical trials involving infection with live parasites have shown some promise in terms of therapeutic benefit to patients with autoimmune inflammatory disease 11, infection with pathogens is clearly not an ideal therapeutic strategy; thus, much recent attention has focused on the idea of developing novel drugs based on the individual helminth molecules (or their antiinflammatory targets) that promote parasite survival by limiting the inflammatory response of the host in a safe manner 9. In this study, we investigated whether ES-62, an immunomodulator secreted by the filarial nematode *Acanthocheilonema viteae* 9, protects against pathology in the MRL/*lpr* mouse model of SLE.

## MATERIALS AND METHODS

### Animal models

Animals were bred and/or maintained in the Biological Services Units at the University of Glasgow and the University of Strathclyde, in accordance with Home Office UK Licences PIL60/9576, PIL60/11671, PIL60/12183, PIL60/12950, PPL60/3580, PPL60/4492, PPL60/4300, and PPL60/3810 and the ethics review boards of these universities. Although lupus-like pathology develops in MRL/Mp mice within 12–18 months, the Fas deficiency in the MRL/Mp^*lpr/lpr*^ (MRL/*lpr*) strain accelerates disease, with these mice developing (within 4 months) high-titer antinuclear antibodies (ANAs), glomerulonephritis, and arthritis-like footpad inflammation as well as the splenomegaly/lymphadenopathy typical of autoimmune lymphoproliferative syndrome 6,12.

Kidney damage, as evidenced by proteinuria, was monitored twice weekly using Multistix (Siemens) and, where indicated, arthritis was scored at the time of culling 13,14. In addition, some mice were tested for renal function as evidenced by serum creatinine and blood urea nitrogen (BUN) levels, using relevant detection kits (Arbor Assays KB02-H1 and K024-H1; Tebu-Bio). The mice were treated twice weekly with phosphate buffered saline (PBS; 100 μl subcutaneously from 7 to 21 weeks of age), purified ES-62 (2 μg in 100 μl PBS subcutaneously from 7 to 21 weeks of age) 15, mouse IgG (Europa Bioproducts) (100 μg in 100 μl PBS intraperitoneally from 7 to 21 weeks of age), or neutralizing anti–IL-22 (AM22.1; 100 μg in 100 μl PBS intraperitoneally from 12 to 21 weeks of age) 16 or anti–IL-17A (MM17F3; 100 μg in 100 μl PBS intraperitoneally from 7 to 12 weeks of age) 17, monoclonal antibodies (kindly provided by Drs. Jean-Christophe Renauld and Jacques Van Snick, Ludwig Institute for Cancer Research, Belgium), or alternatively, with recombinant IL-22 (rIL-22; 1 μg in 100 μl PBS intraperitoneally from 12 to 21 weeks of age) or rIL-17A (PeproTech) (1 μg in 100 μl PBS intraperitoneally from 12 to 21 weeks of age). ES-62 inhibited proteinuria similarly in male and female MRL/*lpr* mice, and proteinuria levels for MRL/*lpr* mice treated with PBS and MRL/*lpr* mice treated with PBS plus IgG were not significantly different. The absence of endotoxin from these reagents was confirmed using an Endosafe Kit (Charles River) 15. Splenic B cells obtained from ES-62– or PBS-treated MRL/*lpr* mice at 21 weeks were purified by negative selection using anti-CD43–labeled magnetic beads (Miltenyi Biotec) (>90% B220+CD3− B2 cells) and transferred into the tail vein of recipient 7-week-old MRL/*lpr* mice (5 × 10^6^ cells in 100 μl sterile PBS). Intravenously administered PBS (100 μl) was used as a control.

### Ex vivo analysis

Blood samples were obtained by cardiac puncture, and red blood cells were lysed prior to flow cytometric analysis. Cells from the spleens or from popliteal, inguinal, axial, and brachial lymph nodes (LNs; 10^6^/ml) were resuspended in RPMI medium containing 2 m*M*l-glutamine, 1 m*M* sodium pyruvate, 100 units/ml penicillin, 100 μg/ml streptomycin, and 1% nonessential amino acids (RPMI complete medium) supplemented with 50 μ*M* 2-mercaptoethanol and 10% heat-inactivated fetal calf serum (all from Invitrogen). Dissection of kidneys and generation of kidney supernatants enriched in interstitial fluid were performed as described previously 18. Following red cell lysis, renal and hematopoietic kidney cells were analyzed by flow cytometry using a gating strategy based on forward scatter versus side scatter exclusion of dead cells/cell debris, exclusion of doublets, and selection of live cells as discriminated using a Live/Dead Fixable Aqua Dead Cell Stain kit (Invitrogen).

For analysis of intracellular cytokine production, cells were incubated with medium or 50 ng/ml phorbol myristate acetate (PMA) plus 500 ng/ml ionomycin (plus 10 μg/ml lipopolysaccharide [*Escherichia coli* O111:B4] for B cell responses) for 1 hour before the addition of 10 μg/ml brefeldin A (Sigma-Aldrich) for a further 5 hours at 37°C with 5% CO_2_ 13. Cells were stained with Live/Dead Fixable Aqua Dead Cell Stain to allow exclusion of dead cells from the analysis following permeabilization, using the solutions and protocols provided by BioLegend. B cell populations 13 were analyzed using the following phenotypic markers: Brilliant Violet 421–conjugated anti-B220, phycoerythrin (PE)–conjugated anti-CD138, Alexa Fluor 700– or PE–Cy7–conjugated anti-CD19; PE-conjugated anti-CD1d; PE–Cy7–conjugated anti-CD23, PE–Cy7–conjugated anti-CD43, PerCP–Cy5.5–conjugated anti-IgD, allophycocyanin (APC)–Cy7–conjugated anti-IgM, APC-conjugated anti-CD16/32, PerCP–Cy5.5–conjugated anti-CD80, APC-conjugated anti-CD206 (all from BioLegend), and eFluor 450–conjugated anti-CD21 (eBioscience) 13.

For the identification of plasmablasts and plasma cells, a dump channel (PerCP) identifying CD11c, CD11b, CD4, CD8, F4/80, and Gr-1 (and CD3, when indicated) markers was used to facilitate exclusion of non-B CD138+ cells 13. Intracellular analysis involved staining with APC-conjugated anti–IL-6, APC-conjugated anti–IL-10 (BioLegend) 13, or anti-myeloid differentiation factor 88 (anti-MyD88) (Abcam) and fluorescein isothiocyanate (FITC)–conjugated goat anti-rabbit IgG (Vector) 14. Data were acquired using BD FACSCalibur and BD LSR II flow cytometers (BD Biosciences) and analyzed using FlowJo software (Tree Star) 13. MyD88 expression in the kidney (30 μg/sample) was additionally assessed by Western blotting using anti-MyD88 (ab2068; Abcam) and densitometric analysis using ImageJ software (National Institutes of Health) 14.

The levels of cytokines (IL-17A [BioLegend], IL-17E and IL-17F [eBioscience], and IL-22 [R&D Systems]) in serum and kidney supernatants were analyzed by enzyme-linked immunosorbent assay 14,19. ANAs were visualized using HEp-2 slides (Antibodies Inc.) and FITC-conjugated anti-mouse IgG (Vector). Quantitative analysis was performed by determining the end point dilutions of serum from individual mice (10^2^–10^5^ log dilutions); the final dilution at which intracellular fluorescence was detectable was recorded. ANA reactivity was visualized using an Axiovert S100 fluorescence microscope (Zeiss).

### Kidney pathology

Kidneys were fixed in formalin (24 hours at 4°C), treated with 30% sucrose (24 hours at 4°C), embedded in Tissue-Tek OCT medium, and snap-frozen in liquid nitrogen. Sections (7 μm) were stained with Harris' hematoxylin and eosin (Sigma-Aldrich) and imaged using an Olympus BX41 camera with Cell software. Hypercellularity was assessed by analyzing 20 glomerular cross-sections per kidney. Deposition of C3a and IgG was detected using rat anti-mouse C3 (11H9: Abcam)/Alexa Fluor 647–conjugated goat anti-rat IgG (Invitrogen) or rabbit anti-mouse IgG (The Jackson Laboratory)/Alexa Fluor 488–conjugated goat anti-rabbit IgG (Invitrogen), respectively, and visualized using an EVOS fluorescence microscope (Life Technologies). Differences between PBS- and ES-62–treated groups were detected using 1:10 and 1:25 dilutions (but not dilutions of ≥1:50) of the primary antibodies.

### Statistical analysis

Proteinuria data were analyzed by two-way analysis of variance with the Bonferroni post hoc test, and experimental data were analyzed by Student's *t*-test. Nonparametric data were analyzed using the Mann-Whitney test.

## RESULTS

### Suppression of proteinuria in MRL/*lpr* mice by ES-62

**Figure 1:**
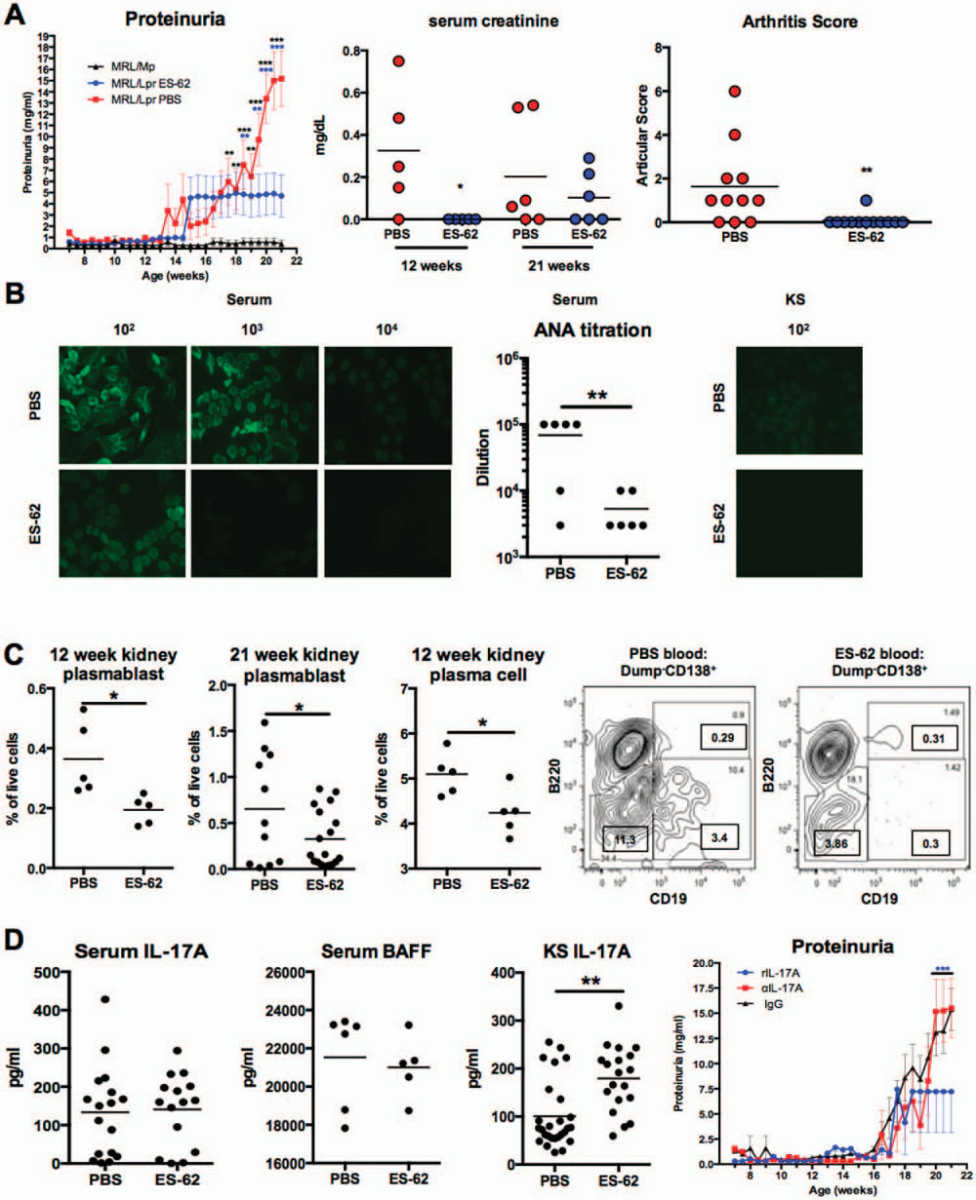
ES-62 suppresses proteinuria and antinuclear antibody (ANA) production in MRL/*lpr* mice. A, Proteinuria was measured twice weekly in male MRL/Mp mice (n = 15) and MRL/*lpr* mice treated with either phosphate buffered saline (PBS) (n = 16) or ES-62 (n = 19), administered subcutaneously twice weekly from 7 to 21 weeks of age. Serum creatinine levels and arthritis articular scores were also determined in some of the individual MRL/*lpr* mice treated with PBS or ES-62 examined for these disease parameters. B, Left, Immunofluorescence imaging of HEp-2 cell staining by ANAs in the serum of PBS-treated and ES-62−treated MRL/*lpr* mice. Original magnification × 63. Middle, ANA levels in the serum of individual PBS-treated (n = 6) and ES-62−treated (n = 6) MRL/*lpr* mice, as measured by end point dilution analysis. Right, Immunofluorescence imaging showing weak detection of ANAs in kidney supernatant (KS) at a dilution of 10^2^. Original magnification × 63. C, Proportions of plasmablast-like CD138+B220^low^CD19+ B cells in the kidneys at 12 and 21 weeks (first and second panels), CD138+B220−CD19− plasma cells in the kidneys at 12 weeks (third panel), and plasmablasts and plasma cells in blood at 12 weeks in mice treated with PBS or ES-62 (fourth and fifth panels). Samples from the relevant treatment groups were pooled. In the fourth and fifth panels, values in the gates are the percentage of Dump−CD138+ cells; values in the boxes are the percentage of live cells. D, Levels of interleukin-17A (IL-17A) and BAFF in serum (first and second panels, respectively), levels of IL-17A in kidney supernatant (third panel) at the time of culling, as measured by enzyme-linked immunosorbent assay, and proteinuria (fourth panel) in MRL/*lpr* mice. Proteinuria was measured twice weekly in mice that received twice-weekly intraperitoneal injections of mouse IgG (100 μg in 100 μl PBS from 7 to 21 weeks, n = 16) or anti–IL-17A (100 μg in 100 μl PBS from 7 to 12 weeks, n = 8) or recombinant IL-17A (rIL-17A; 1 μg in 100 μl PBS from 12 to 21 weeks, n = 6). Values for proteinuria are the mean ± SEM and in A are collated from 3 independent experiments. In A (second and third panels), B (middle panel), C (first, second, and third panels), and D (first, second, and third panels), each symbol represents an individual mouse; bars show the mean. ∗ = *P* < 0.05; ∗∗ = *P* < 0.01; ∗∗∗ = *P* < 0.001. Black asterisks indicate PBS-treated MRL/*lpr* mice versus MRL/Mp mice. Blue asterisks indicate PBS-treated versus ES-62–treated MRL/*lpr* mice or rIL-17A–treated versus murine IgG–treated mice.

Proteinuria, a surrogate for glomerular vascular permeability, inflammation, and kidney damage, was detected in MRL/*lpr* mice, but not in MRL/Mp mice, by 16 weeks of age and increased thereafter, indicating that progressive kidney damage was occurring (Figure 1A). Treatment of the MRL/*lpr* mice with ES-62 reduced the level of proteinuria (Figure 1A) and the associated incidence of disease (>3 mg/ml protein: 0% in MRL/Mp mice, 100% in PBS-treated MRL/*lpr* mice, and 22.2% in ES-62–treated MRL/*lpr* mice). Moreover, additional parameters of renal function were also tested in some mice (proteinuria at 21 weeks: 20 mg/ml in all PBS-treated mice and mean ± SEM 0.98 ± 0.36 mg/ml in ES-62–treated mice), and this showed that ES-62 reduced serum creatinine levels both prior to and during established proteinuria (12 weeks and 21 weeks, respectively), although at 21 weeks, the reduction did not reach significance (Figure 1A).

In contrast, ES-62 did not significantly reduce BUN levels (data not shown), but this presumably reflected that these levels were still in the normal range (∼35 mg/dl), because typically these levels do not become elevated in MRL/*lpr* mice until ∼24–32 weeks of age 20–22. However, both the severity of arthritis (Figure 1A) and the incidence of arthritis (72.7% in the PBS-treated mice and 8.3% in the ES-62–treated mice) were suppressed in the ES-62–treated MRL/*lpr* mice examined. Moreover, although systematic survival analysis was precluded due to ethical constraints, exposure to ES-62 promoted the survival of MRL/*lpr* mice (66.7% of PBS-treated mice and 92.9% of ES-62–treated mice) over the 21-week time course of the experiments.

### ES-62–induced suppression of ANA production in MRL/*lpr* mice

ES-62 did not significantly modulate the levels of total IgG1, IgG2a, or IgM in the serum of MRL/*lpr* mice (data not shown). In contrast, ES-62 inhibited the production of ANAs, as measured in the serum of MRL/*lpr* mice, both prior to (12 weeks; data not shown) and during established disease (21 weeks). Similarly, the levels of ANA weakly detected in the kidney supernatants were also reduced following exposure to ES-62 (Figure 1B). Consistent with this suppression of pathogenic autoantibody production, the numbers (data not shown) and proportions of the plasmablast-like CD138+B220^low^CD19+ B cells that may represent short-lived plasma cells 13 were reduced in the kidneys of mice exposed to ES-62, at both 12 weeks and 21 weeks (Figure 1C). These cells are associated with disease flares in patients with SLE and are the likely source of pathogenic anti–double-stranded DNA (anti-dsDNA) IgG2a and IgG3 autoantibodies 23. In contrast, the percentage of long-lived CD138+B220−CD19− plasma cells 13, which have been reported to be responsible for producing anti-RNA and anticardiolipin antibodies 23, was reduced in the kidneys prior to (Figure 1C) but not during established disease (mean ± SEM 1.8 ± 0.6% in PBS-treated mice and 2.4 ± 0.6% in ES-62–treated mice). At 21 weeks, however, the proportions of both plasmablasts and plasma cells were reduced in the blood of ES-62–treated mice relative to PBS-treated mice (Figure 1C).

### Association between ES-62–induced suppression of B cell responses in MRL/*lpr* mice and resetting the balance between effector and regulatory B cells

To address the mechanisms underpinning the suppression of ANA production and the reduced levels of CD138+B220^low^CD19+ plasmablast-like B cells, we first investigated the effect of in vivo exposure to ES-62 on BAFF and IL-17 expression, because these cytokines have been proposed to synergize and promote (auto)antibody production 4,5. This investigation revealed that ES-62 did not suppress the levels of either cytokine in the serum or kidney supernatants of MRL/*lpr* mice (mean ± SEM BAFF levels in kidney supernatants 4,823 ± 333 pg/ml in PBS-treated mice and 5,084 ± 1,543 pg/ml in ES-62–treated mice) (Figure 1D), nor did the parasite product decrease the levels in kidney supernatants of IL-17E (mean ± SEM 949 ± 127 pg/ml in PBS-treated mice and 1,067 ± 153 pg/ml in ES-62–treated mice) or IL-17F (333 ± 61 pg/ml in PBS-treated mice and 433 ± 104 pg/ml in ES-62–treated mice), the latter of which was recently correlated with disease activity in SLE 24. Moreover, neutralizing anti–IL-17 antibodies did not block ANA production (data not shown) or development of proteinuria (Figure 1D). Indeed, ES-62 tended to promote IL-17 production in the kidney during established proteinuria, and consistent with this, administration of rIL-17 from 12 weeks onward partially suppressed proteinuria (incidence 40%) (Figure 1D).

**Figure 2:**
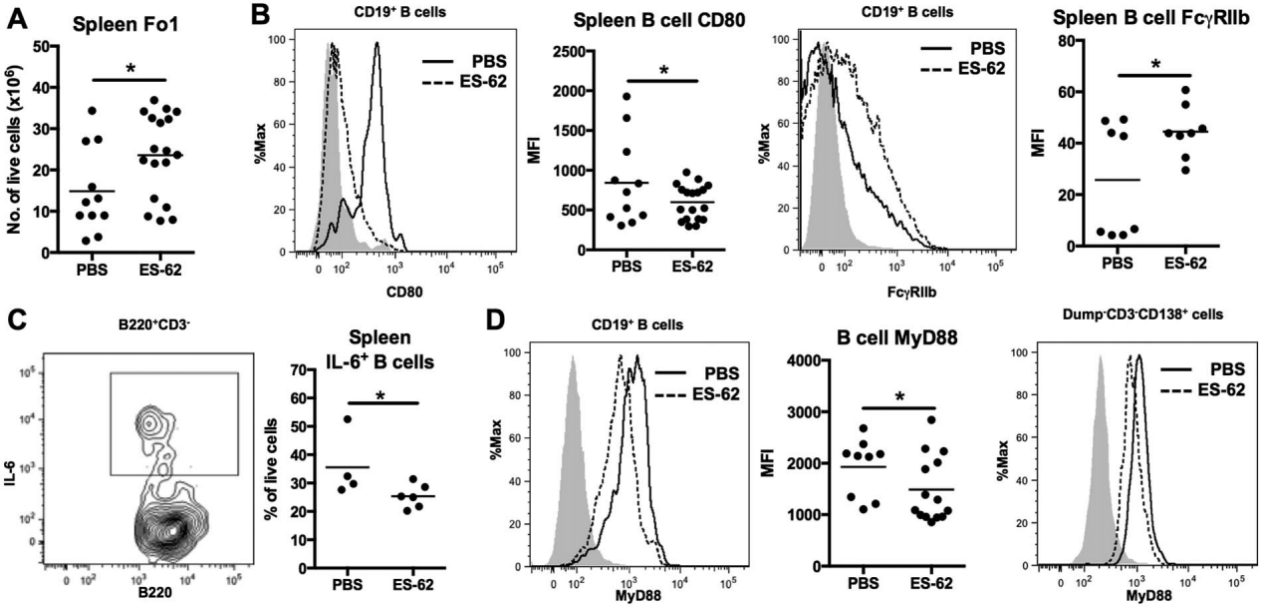
ES-62 modulates effector B cells by targeting myeloid differentiation factor 88 (MyD88). A, Total numbers of splenic follicular 1 (Fo1) B cells (CD19+CD93−CD21^intermediate^CD23+IgD^high^IgM^low^) in individual 21-week-old PBS- and ES-62–treated MRL/*lpr* mice, as determined by flow cytometry. B, Expression of CD80 (first and second panels) and Fcγ receptor IIb (FcγRIIb) (third and fourth panels) on CD19+ splenic B cells from PBS- and ES-62–treated MRL/*lpr* mice. C, Expression of IL-6–producing splenic B cells from MRL/*lpr* mice following in vivo exposure to PBS or ES-62. D, Intracellular levels of MyD88 in CD19+ and Dump–CD3–CD138+ B cells from PBS- and ES-62–treated MRL/*lpr* mice. In A (first panel), B (second and fourth panels), C (second panel), and D (middle panel), each symbol represents an individual mouse; bars show the mean. ∗ = *P* < 0.05. MFI = mean fluorescence intensity (see Figure 1 for other definitions).

Mice treated with ES-62 displayed increased total numbers of splenic CD19+ B cells (mean ± SEM 61.5 ± 7.5 × 10^6^ in PBS-treated mice [n = 11] and 90.02 ± 9.2 × 10^6^ in ES-62–treated mice [n = 18]) and follicular 1 B cells (CD19+CD93−CD21^intermediate^CD23+IgD^high^IgM^low^) (Figure 2A) but not T cells (results not shown), perhaps suggesting that the reduced plasmablast differentiation reflected induction of a hyporesponsive phenotype of B cells. Consistent with this, expression of CD80 on splenic B cells was down-regulated, while that of Fcγ receptor IIb was up-regulated (Figure 2B) in ES-62–treated MRL/*lpr* mice. Furthermore, following ex vivo stimulation, the levels of IL-6–producing splenic B cells, which are proposed to be an important driver of autoimmunity in mice 25, were reduced by in vivo exposure to ES-62 (Figure 2C). This was reflected by a reduction in the IL-6 messenger RNA levels (mean ± SEM relative quantity value 0.67 ± 0.13) observed in splenic CD19+CD3− B cells purified from ES-62–treated MRL/*lpr* mice when normalized to those from PBS-treated mice.

Similarly, such purified splenic B cells from ES-62–treated mice produced less interferon-γ than those from PBS-treated control mice (mean ± SEM 284 ± 104 versus 540 ± 51 pg/ml) in ex vivo cultures. However, surface expression of IgD and IgM was not modulated (data not shown); thus, ES-62 does not simply induce anergy resulting from down-regulation of the B cell receptor. Intriguingly, given the abrogation of ANA responses reported in MRL/*lpr* mice with MyD88-deficient B cells 26, B cells, including CD138+ B cells (Figure 2D) from ES-62–treated MRL/*lpr* mice, exhibited reduced levels of MyD88.

**Figure 3:**
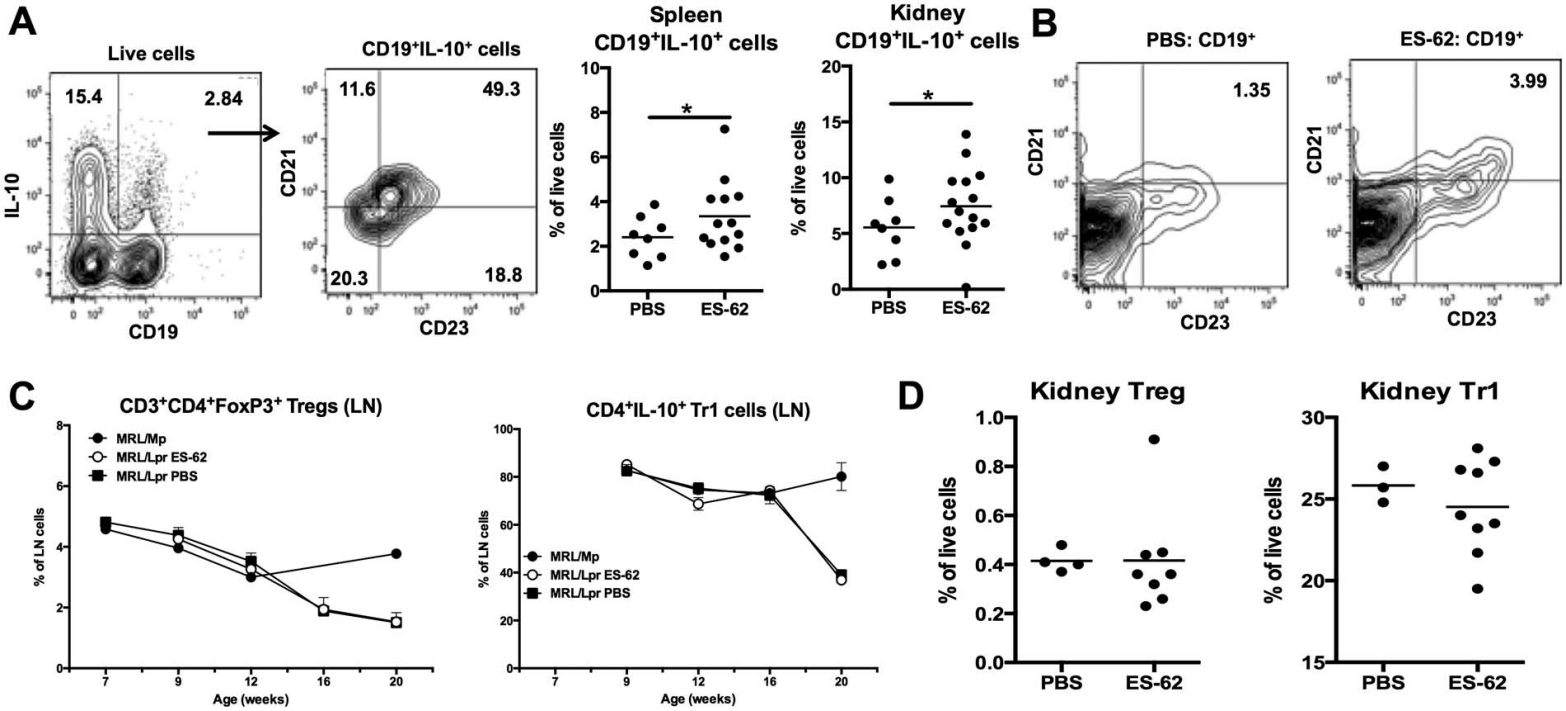
ES-62 induces IL-10–producing B cells but not T cells. A, IL-10 production by spleen and kidney cells from PBS- and ES-62–treated 21-week-old MRL/*lpr* mice stimulated ex vivo, as determined by flow cytometry. B, Proportions of CD19+CD21+CD23+ B cells in the blood of PBS- and ES-62–treated MRL/*lpr* mice at 21 weeks. C, Proportions of FoxP3+ Treg cells (left) and IL-10+ Tr1 cells (right) in the lymph nodes (LNs) of MRL/Mp mice (n = 3), ES-62–treated MRL/*lpr* mice (n = 3–5), and PBS-treated MRL/*lpr* mice (n = 5) at all time points. Values are the mean ± SEM. D, Levels of Treg cells and Tr1 cells in the kidneys of MRL/*lpr* mice at 21 weeks. In A (third and fourth panels) and D, each symbol represents an individual mouse; bars show the mean. ∗ = *P* < 0.05. See Figure 1 for other definitions.

In contrast, the levels of B cells with the capacity to produce IL-10, particularly CD19+CD21+CD23+ B cells analogous to those proposed to exhibit regulatory function in MRL/*lpr* mice and SLE 27,28, were increased in the spleen and kidney in ES-62–treated mice (Figure 3A). Moreover, the levels of CD19+CD21+CD23+ B cells were increased in the blood of ES-62–treated mice (Figure 3B). Such “regulatory” B cells have been reported to mediate their protective effects, at least in part, via the induction of Treg cells, particularly IL-10–producing CD4+ T (Tr1) cells, in MRL/*lpr* mice 27,28. Consistent with the proposed protective role of Treg cells in SLE 29, the proportion of FoxP3+CD4+ Treg cells and IL-10+CD4+Tr1 cells in the LNs of MRL/*lpr*, but not MRL/Mp, mice declined with age (although the absolute numbers increased), with kinetics that correlated with the initiation and progression of proteinuria. Treatment with ES-62, however, did not increase the levels of Treg or Tr1 cells in the LNs (Figure 3C), spleens (data not shown), or kidneys (Figure 3D) of MRL/*lpr* mice.

### ES-62–induced protection against kidney damage correlates with antagonism of IL-22 responses and is mimicked by transfer of B cells from ES-62–treated MRL/*lpr* mice

**Figure 4:**
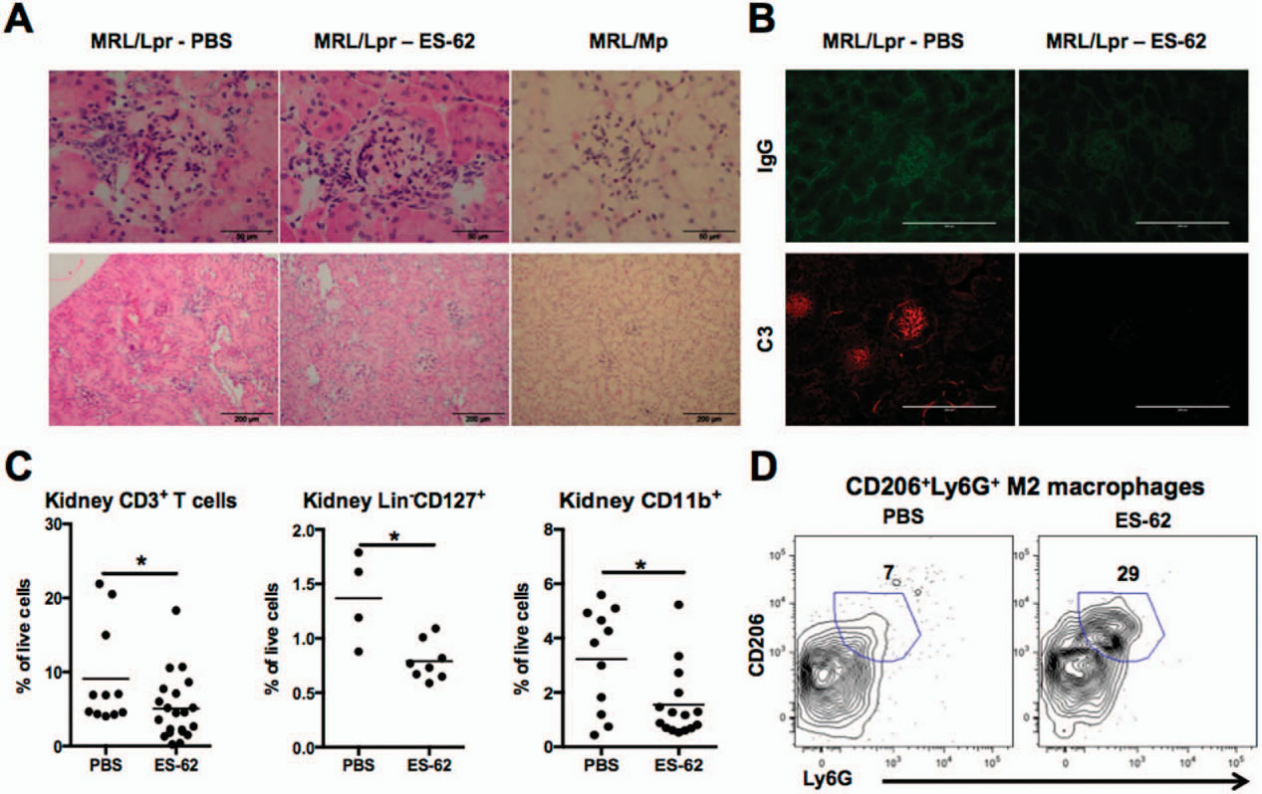
ES-62 modulates cellular infiltration and deposition of IgG and C3 in the kidney. A, Glomerular hypercellularity (top) and cellular infiltration (bottom) in hematoxylin and eosin−stained kidney sections from 21-week-old MRL/Mp and MRL/*lpr* mice treated with PBS or ES-62. Original magnification × 40 (top); × 10 (bottom). B, Deposition of IgG and C3 in the kidneys of MRL/*lpr* mice treated with PBS or ES-62, as detected using rabbit anti-mouse IgG/Alexa Fluor 488−conjugated goat anti-rabbit IgG and rat anti-mouse C3/Alexa Fluor 647−conjugated goat anti-rat IgG, respectively. Original magnification × 40. C, Proportions of CD3+ T cells, Lin−CD127+ innate lymphoid cells, and CD11b+ cells in the kidneys of MRL/*lpr* mice treated with PBS or ES-62. Each symbol represents an individual mouse; bars show the mean. D, Levels of CD206+Ly-6G+ M2 macrophages in the kidneys of MRL/*lpr* mice treated with PBS or ES-62. The values shown represent the percentage of cells in the CD206+Ly-6G+ gate; when indicated, however, these were further analyzed as F4/80+CD11c− cells as a proportion of live cells. ∗ = *P* < 0.05. See Figure 1 for definitions.

Perhaps surprisingly, given the striking inhibition of proteinuria, histologic analysis of kidneys from ES-62–treated MRL/*lpr* mice (Figure 4A) did not reveal any substantial modulation of glomerular hypercellularity, as confirmed by counting cells within individual glomeruli (mean ± SEM 63.6 ± 4.8 in PBS-treated mice [n = 26] and 64.4 ± 7.4 in ES-62–treated mice [n = 18]). Exposure to ES-62 did, however, reduce IgG and C3 deposition in the kidneys (Figure 4B) and modulated the phenotype of the infiltrating cell population, selectively reducing the proportion of CD3+ T cells, Lin− CD127+ innate lymphoid cells (ILCs), and CD11b+ cells (Figure 4C) while increasing the levels of antiinflammatory F4/80^high^CD11c−CD206+Ly-6G+ M2 macrophages (Figure 4D) (mean ± SEM 2 ± 0.4% of live cells in PBS-treated mice and 5.5 ± 2.5% of live cells in ES-62–treated mice) associated with protection, which appear to be depleted in SLE 30.

**Figure 5:**
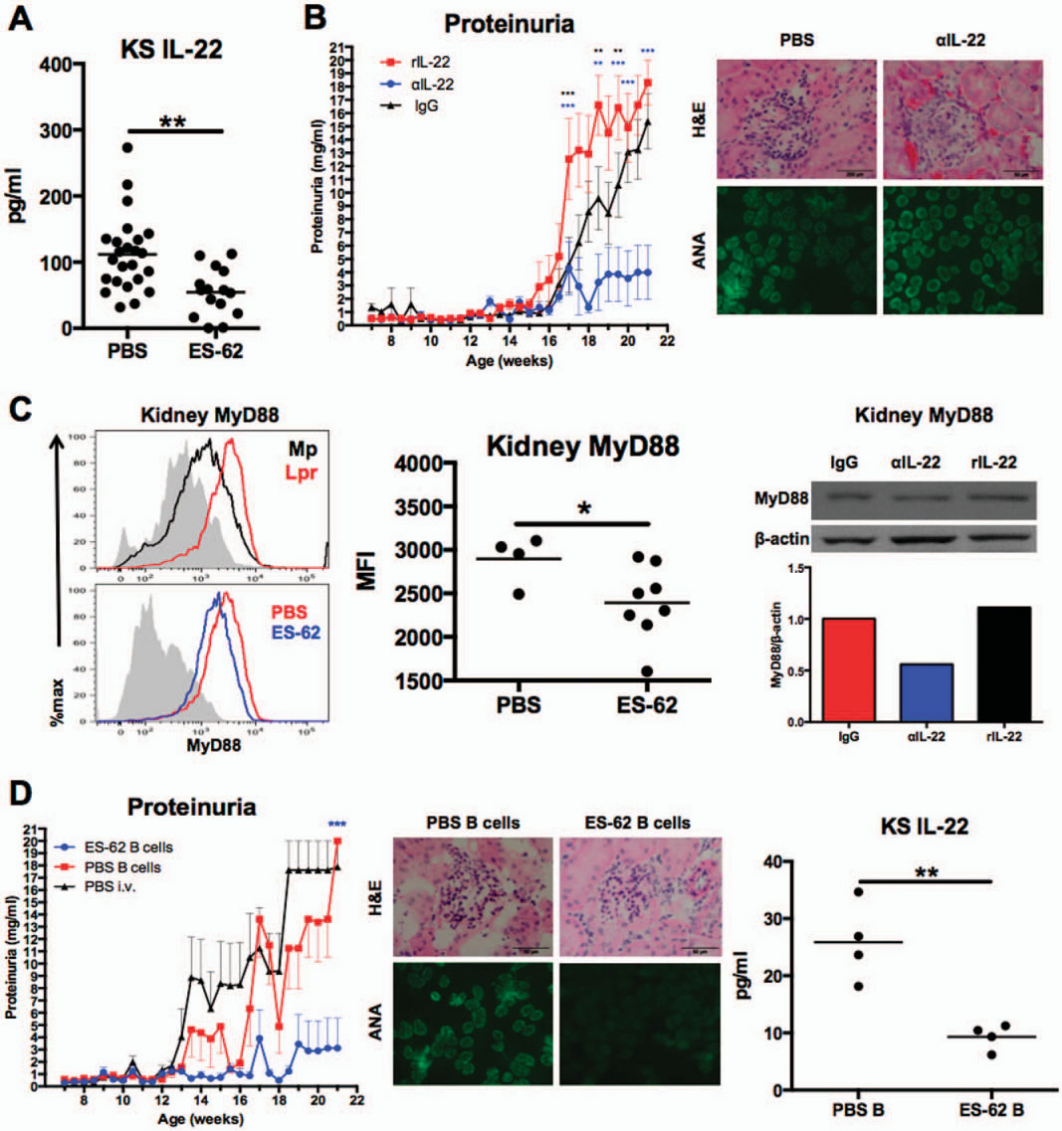
ES-62 suppresses pathogenic IL-22 production and myeloid differentiation factor 88 (MyD88) expression in the kidney. A, IL-22 levels in kidney supernatant derived from PBS- and ES-62−treated MRL/*lpr* mice, as measured by enzyme-linked immunosorbent assay. B, Left, Proteinuria in MRL/*lpr* mice that received twice-weekly intraperitoneal injections of rIL-22 (1 μg in 100 μl PBS from 12 to 21 weeks of age, n = 10), anti−IL-22 (100 μg in 100 μl PBS from 12 to 21 weeks of age, n = 9), or mouse IgG (100 μg in 100 μl PBS from 7 to 21 weeks of age, n = 16). The IgG data are the same as those shown in Figure 1D, as the matched IL-17 and IL-22−modulated groups were analyzed in parallel cohorts to promote the 3 Rs (replace, reduce, refine). Right, Glomerular hypercellularity in hematoxylin and eosin (H&E)−stained kidney sections (top) and ANA production in serum (10^2^ dilution) (bottom) from MRL/*lpr* mice treated with PBS or anti−IL-22. Original magnification × 40 (top); × 63 (bottom). C, Left, MyD88 expression in kidney cells from MRL/Mp and MRL/*lpr* mice at 12 weeks of age (top) and from PBS- and ES-62−treated MRL/*lpr* mice at 21 weeks of age (bottom), as determined by flow cytometric analysis. Middle, MyD88 expression in the kidneys of individual PBS- or ES-62−treated MRL/*lpr* mice. Right, Western blots showing MyD88 levels in kidney protein lysates derived from MRL/*lpr* mice treated with IgG, anti−IL-22, or rIL-22 (top), and densitometric analysis of MyD88/β-actin expression, normalized to IgG control (bottom). D, Proteinuria (left), glomerular hypercellularity and ANA production (middle), and IL-22 expression (right) in recipient MRL/*lpr* mice in which splenic B cells harvested from MRL/*lpr* mice were transferred. Purified splenic CD43− B cells from 21-week-old MRL/*lpr* mice treated with ES-62 (n = 4) or PBS (n = 4) were transferred (5 × 10^6^ cells pooled in 100 μl sterile PBS) into the tail veins of 7-week-old MRL/*lpr* mice; intravenous (IV) PBS was used as a control. Proteinuria in the recipient mice (6 received PBS, 8 received PBS-treated B cells, and 8 received B cells from ES-62–treated mice) was measured twice weekly. Kidney sections were stained with H&E for analysis of glomerular hypercellularity, and serum analyzed for ANA production. IL-22 was measured in the kidneys of mice from one of these experiments. Values for proteinuria are the mean ± SEM (n = number of relevant individual mice pooled from 2 independent experiments). In A, C (middle), and D (right), each symbol represents an individual mouse; bars show the mean. ∗ = *P* < 0.05; ∗∗ = *P* < 0.01; ∗∗∗ = *P* < 0.001. MFI = mean fluorescence intensity (see Figure 1 for other definitions).

The cytokine IL-22, which can promote barrier integrity and wound repair 31, was recently reported to stimulate kidney regeneration after acute injury by acting on tubular epithelial cells 32. In the current study, however, ES-62 suppressed the levels of IL-22 in kidney supernatants (Figure 5A). Thus, as IL-23 promotes IL-22 responses, and because of the increasing recognition of pathogenic roles for IL-22 in autoimmune disorders 33,34 including SLE 35–38, we investigated whether IL-22 production in the kidney was associated with SLE pathogenesis. Administration of rIL-22 significantly accelerated and exacerbated the development of proteinuria. In contrast, neutralization of this cytokine suppressed proteinuria, although treatment with anti–IL-22 did not prevent glomerular hypercellularity or ANA production (Figure 5B). Exposure of MRL/*lpr* mice to ES-62 plus anti–IL-22 resulted in no significant differences between this combination treatment and protocols with either anti–IL-22 or ES-62 plus IgG alone (data not shown). Instead, although expression of MyD88 was up-regulated in kidney cells from MRL/*lpr* mice relative to MRL/Mp mice, it was reduced in kidney cells from ES-62–treated MRL/*lpr* mice and anti–IL-22–treated MRL/*lpr* mice, and rIL-22 appeared to maintain (if not substantially increase) MyD88 levels (Figure 5C). Collectively, these data are consistent with the notion that IL-22 plays a pathogenic role in promoting MyD88-dependent inflammation and vascular barrier permeability in the MRL/*lpr* mouse and suggest that this cytokine activity may be targeted by ES-62 to mediate some of its protective effects in the kidney.

Finally, in order to confirm that the protection against proteinuria afforded by the helminth product was attributable to ES-62 resetting effector B cell responses, we investigated the effect of adoptively transferring purified splenic B cells harvested from MRL/*lpr* mice treated with either PBS or ES-62 into recipient 7-week-old MRL/*lpr* mice. Strikingly, the transfer of B cells from ES-62–treated MRL/*lpr* mice was sufficient to provide significant protection against the development of proteinuria in the recipient mice despite, as was observed with ES-62, no substantial improvement in glomerular hypercellularity (Figure 5D). However, transfer of B cells from ES-62–treated mice did suppress pathogenic ANA production (Figure 5D) (mean ± SEM reciprocal end point dilutions 68,500 ± 19,956 in mice treated with intravenous PBS, 55,000 ± 25,981 in mice that received B cells from MRL/*lpr* mice treated with PBS, and 5,500 ± 5,196 in mice that received B cells from MRL/*lpr* mice treated with ES-62) and reduced IL-22 levels in kidney supernatants (Figure 5D), indicating that modulation of IL-22 responses is secondary to ES-62–mediated resetting of the balance between effector and regulatory B cells.

## DISCUSSION

**Figure 6:**
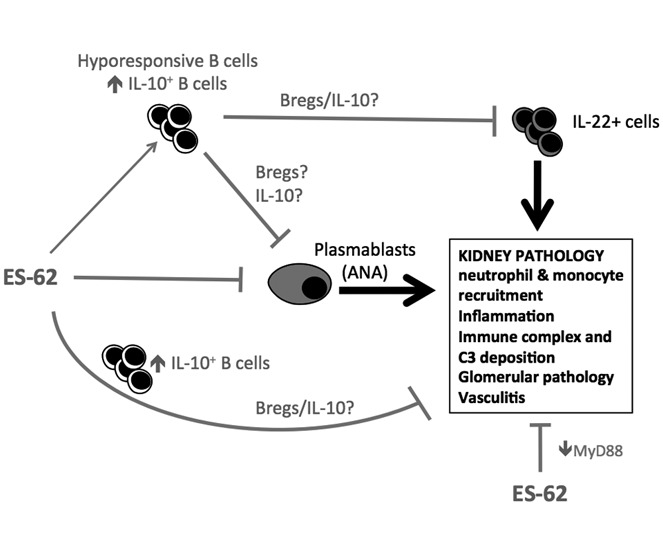
Schematic representation of the action of ES-62. ES-62 desensitizes hyperresponsive B cells in MRL/*lpr* mice, resulting in a lower level of plasmablasts and, consequently, suppression of antinuclear antibody (ANA) production and IgG and C3a deposition in the kidney. This population of hyporesponsive effector B cells has a higher frequency of B cells that can potentially reset the balance between effector cells and regulatory cells via the production of interleukin-10 (IL-10) and/or other regulatory B (Breg) cell mechanisms. In addition to reducing the levels of pathogenic ANAs, this hyporesponsive phenotype is associated with a reduction in pathogenic IL-22 responses in the kidney, which is reflected, at least in part, by down-regulation of myeloid differentiation factor 88 (MyD88) expression in kidney cells. ES-62 may also act directly to down-regulate MyD88 in kidney cells and in this way act to desensitize the pathogenic cross-talk among Toll-like receptors, Fc receptors, and complement receptors that results in glomerular vascular permeability, inflammation, and kidney damage.

ES-62 significantly suppresses pathogenic ANA production (but not total IgG or IgM production) and consequent deposition of IgG and C3a in the kidneys of MRL/*lpr* mice. In addition, ES-62 reduces IL-22 levels and modulates the phenotype of the kidney cellular infiltrate, effects that collectively result in suppressed development of proteinuria (Figure 6), a biomarker of the kidney inflammation and damage that are the major causes of mortality in the MRL/*lpr* mouse model of SLE 12. ES-62 appears to prevent ANA generation by down-regulating the expression of B cell MyD88; the notion that this homeostatic regulation of effector B cell responsiveness by the helminth product is relevant to the observed protection is validated by studies in which purified splenic B cells from ES-62–treated MRL/*lpr* mice similarly suppress ANA production and also development of proteinuria in recipient MRL/*lpr* mice. Moreover, and consistent with our findings, studies by other investigators using the MRL/*lpr* 26 and Lyn^−/−^ 39 mouse models of SLE have shown that complete deletion of MyD88 in B cells is sufficient to abrogate ANA production (but not that of total IgG), proteinuria, and glomerulonephritis, and as with ES-62, reduces the number of plasmablasts while increasing the numbers of follicular and total splenic B cells.

The IL-23/IL-17A axis has been proposed to play a pathogenic role in SLE 4,5, but ES-62 did not suppress IL-17 production, and neutralizing antibodies to this cytokine did not abrogate disease progression. Although anti–IL-17 treatment has been shown to suppress the levels of anti-dsDNA antibodies and proteinuria in the MRL/*lpr* mouse model, this occurred during the initiation phase (∼12 weeks) 6, and, consistent with our observations that the number of plasma cells decreased at this stage, ES-62 suppressed the levels of IL-17A–producing CD4+ and γ/δ T cells in LNs at 9 weeks (data not shown). Moreover, although levels of IL-17A have been widely shown to be elevated in patients with SLE, only a few studies have shown that IL-17A levels correlate with the SLE Disease Activity Index (SLEDAI) 40–48, with slightly more studies concluding either that IL-17A levels do not correlate with the SLEDAI or that these levels are actually inversely correlated with the disease score 49–60.

Of note, IL-23 can also promote IL-22 responses 61, a finding that perhaps reconciles some of these contradictory data relating to the role of the inflammatory IL-23/IL-17 axis in SLE. Pertinently, we observed that IL-17 and IL-22 are produced by distinct populations of cells in the MRL/*lpr* mouse, with typically at the time of culling only 1–2% of IL-22+ cells in LNs producing IL-17 in response to ex vivo stimulation with PMA/ionomycin. Moreover, although exposure to ES-62 results in significantly lower levels of IL-22+ but not IL-17+ CD3+B220+CD4−CD8− (double-negative) T cells, it induces higher levels of IL-17+ but not IL-22+ ILCs, suggesting that targeting of particular cellular sources of IL-17 and IL-22 allows ES-62 to modulate the distinct, potentially counterregulatory, effector functions of these cytokines in the MRL/*lpr* mouse (data not shown).

Thus, while not ruling out the possibility that IL-17A may promote initiation of pathogenesis, our data clearly suggest that this cytokine can resolve inflammation during established disease, a hypothesis that is consistent with the proposed dual roles of IL-17 in initiating and resolving kidney disease in experimental crescentic glomerulonephritis 62. In contrast, and consistent with its role in linking the regulation of inflammatory responses and barrier tissue homeostasis 31,34, IL-22 appears to promote pathogenic effector cell mechanisms in the kidney. Thus, although a disease-causing role for IL-22 has yet to be established unequivocally in SLE, our data resonate with reports that this cytokine is associated with pathogenesis 36,37 and may be predictive of specific pathologies in certain patients with SLE 35.

Although deletion of MyD88 signaling in B cells is sufficient to abrogate lupus nephritis in the MRL/*lpr* and Lyn^−/−^ mouse models 26,39, MyD88 deficiency in dendritic cells (DCs) can also confer some protection and highlights the cooperation between B cells and DCs 26,63, and potentially other (nonhematopoietic) cells, in the development of SLE-like pathologies. Interestingly, therefore, MyD88-dependent cooperation between myeloid and endothelial cells was recently proven to be key to the promotion of the vascular inflammation and atherosclerosis associated with metabolic syndrome 64. Thus, because MyD88 is likely to be a key player in integrating cross-talk among TLR, FcR, and complement receptors, in concert with the reduction in IgG and C3a deposition, our finding that ES-62 and anti–IL-22 down-regulate MyD88 expression in kidney cells provides an effective mechanism for protecting against kidney inflammation. In addition, and relating to the IL-22–mediated regulation of barrier function and vascular inflammation alluded to above, IL-1β was recently shown to act on endothelial cells to stimulate an NF-κB–independent, MyD88/ADP ribosylation factor (ARF) nucleotide binding site opener (ARNO)/ARF 6–dependent pathway of vascular permeability that contributes to vasculitis in autoimmune disease 65. Therefore, disruption of the cooperative interplay resulting from partial down-regulation of MyD88 signaling in B cells and kidney cells would act to break the persistent cycle of inflammation and vascular permeability resulting in kidney damage in SLE without fully immunosuppressing the host.

The ability of ES-62 to suppress pathogenic B cell and effector cell responses appears to be associated with a homeostatic resetting of the effector cell–to–regulatory cell balance, as indicated by the reduction in the frequency of pathogenic plasmablasts and the increased number of CD19+CD23+CD21+ B cells with the capacity to produce IL-10, a phenotype reminiscent of the regulatory B cells proposed to be defective in MRL/*lpr* mice 28 and SLE 27. Interestingly, there is increasing evidence that the dampening of inflammatory responses by some helminth products may reflect, at least in part, activation of regulatory B (“Breg”) cell function 66,67. Indeed, patients with multiple sclerosis who have helminthic infection exhibit less severe disease, and such protection appears to be associated with elevated levels of IL-10–producing B cells 68. Moreover, in a mouse model of asthma, IL-10–producing B cells induced by the trematode helminth *Schistosoma mansoni* suppressed disease 69,70, and in addition, B cells from mice infected with the gastrointestinal nematode *Heligmosomoides polygyrus* protected against the development of both allergic airway inflammation and autoimmune inflammation in an experimental model of autoimmune encephalomyelitis 71. Nevertheless, recent genetic studies suggest that regulatory B cells do not counterregulate pathogenic effector B cell responses in the MRL/*lpr* mouse in an IL-10–dependent manner 72, and although these data may reflect their dysfunctional phenotype and loss in this mouse strain, our preliminary in vitro data also suggest that while B cells from ES-62–treated MRL/*lpr* mice inhibit T cell responses, they may do so in an IL-10–independent manner.

These findings reflect the increasing recognition that regulatory B cells can exploit a variety of mechanisms to limit chronic inflammation 73, and in any case, expanded populations of such regulatory B cells can transfer protection in MRL/*lpr* mice 27,28, suggesting that regardless of their mode of action, they could be exploited therapeutically. To date, however, therapies aimed at targeting effector B cells and/or resetting the balance between effector and regulatory cells have been disappointing in clinical trials in SLE; therefore, the need remains for the development of new and safer therapies to achieve this. Thus, exploiting the targets identified by parasitic helminths, which appear to have evolved such homeostatic actions as regulating proinflammatory MyD88 signaling in several cell types including B cells, as a general and safe mechanism to dampen hyperinflammatory responses may provide an alternative blueprint for the development of novel biologic agents or drugs to treat SLE.

## AUTHOR CONTRIBUTIONS

All authors were involved in drafting the article or revising it critically for important intellectual content, and all authors approved the final version to be published. Dr. M. M. Harnett had full access to all of the data in the study and takes responsibility for the integrity of the data and the accuracy of the data analysis.

**Study conception and design.** Rodgers, McGrath, Pineda, W. Harnett, M. M. Harnett.

**Acquisition of data.** Rodgers, McGrath, Pineda, Al-Riyami, Rzepecka, Lumb.

**Analysis and interpretation of data.** Rodgers, McGrath, Al-Riyami, Lumb, W. Harnett, M. M. Harnett.
